# Biomechanical Finite Element Analysis of Two Types of Short-Angled Implants Across Various Bone Classifications

**DOI:** 10.3390/ma17235680

**Published:** 2024-11-21

**Authors:** Mario Ceddia, Tea Romasco, Nilton De Bortoli, Bruno Freitas Mello, Adriano Piattelli, Eitan Mijiritsky, Natalia Di Pietro, Bartolomeo Trentadue

**Affiliations:** 1 Department of Mechanics, Mathematics and Management, Politecnico di Bari University, 70125 Bari, Italy; marioceddia1998@gmail.com (M.C.); bartolomeo.trentadue@poliba.it (B.T.); 2 Department of Medical, Oral and Biotechnological Sciences, “G. d’Annunzio” University of Chieti-Pescara, 66100 Chieti, Italy; tea.romasco@unich.it; 3 Center for Advanced Studies and Technologies (CAST), “G. d’Annunzio” University of Chieti-Pescara, 66100 Chieti, Italy; 4 Department of Oral Implantology, Associação Paulista dos Cirurgiões Dentistas-APCD, São Bernardo do Campo 02011-000, Brazil; bortoliusp@gmail.com; 5 Department of Periodontics and Implant Dentistry, University of Vale do Itajaí, Itajaí 88302-901, Brazil; brunofmellofloripa@gmail.com; 6 School of Dentistry, Saint Camillus International University of Health and Medical Sciences, 00131 Rome, Italy; apiattelli51@gmail.com; 7 Facultad de Medicina, UCAM Universidad Católica San Antonio de Murcia, 30107 Murcia, Spain; 8 Department of Head and Neck Surgery and Maxillofacial Surgery, Tel-Aviv Sourasky Medical Center, School of Medicine, Tel-Aviv University, Tel Aviv 64239, Israel; mijiritsky@bezeqint.net; 9 Goldschleger School of Dental Medicine, Faculty of Medicine, Tel-Aviv University, Tel Aviv 39040, Israel

**Keywords:** finite element analysis (FEA), dental implants, dental stress analysis, short implants, angled implants, inclined abutments

## Abstract

The aim of this finite element analysis (FEA) was to investigate the distribution of von Mises stress within dental implant components, as well as trabecular and cortical bone. The study considered various bone qualities that influence cortical thickness in contact with the implant, specifically examining cortical thicknesses of 0.5, 1.5, and 3 mm, corresponding to Bergkvist’s classifications IV, III, and II, respectively. A simplified 3D model of the bone was developed for the analysis. Two short implants were inserted into the model: one with a 30° inclined abutment (IA) and another positioned at a 30° angle featuring a straight abutment (II). A vertical force (120 N) was applied to the upper surface of the abutments. FEA software was employed to assess the stresses on the peri-implant tissues and the implants. The findings indicated that a reduction in cortical bone thickness results in an increase in stress within the cortical bone. For IA, the stresses recorded 32.56, 56.12, and 96.14 MPa for cortical thicknesses of 3, 1.5, and 0.5 mm, respectively. Conversely, II exhibited increased stresses across all bone qualities (52.32, 76.15, and 126.32 MPa for the same cortical thicknesses). It is advisable to avoid II in cases of poor bone quality and thin cortical due to the heightened risk of overload-induced bone resorption; however, it may be preferable to use IA in scenarios involving good bone quality and thicker cortical.

## 1. Introduction

Teeth are firmly anchored within the alveolar bone, which possesses mechanical properties that enable it to withstand the forces generated during chewing [1,2,3,4,5,6]. The alveolar bone exhibits the capacity to adapt to mechanical stresses through a process known as remodeling. According to Wolff’s law, the bone responds adaptively to external forces [7]. Subjecting the bone to mechanical stimuli is essential for maintaining its structure and density. The loss of a tooth, however, results in the absence of stimulation to the residual bone, thereby causing a decrease in density and structural integrity, which can lead to a reduction in width, height, and thickness. This reduction in bone volume may result in atrophic ridges, complicating the use of traditional prosthetics [7,8].

Bone augmentation represents a widely utilized technique in dentistry, which may employ either osteoinductive or osteoconductive materials to promote bone regeneration and facilitate the placement of standard dental implants [9]. Short implants have emerged as a viable treatment option for patients with atrophic or resorbed bone, whether partially or fully edentulous. The definition of short implants varies among authors; some define them as implants measuring less than 10 mm in length, while others consider implants shorter than 7 mm to be classified as short [10,11,12,13]. In 2016, a European agreement established that short implants are classified as those measuring 8 mm or less, while ultra-short implants are categorized as those measuring less than 6 mm [14]. Numerous studies have documented the success and survival rates associated with short implants, which historically exhibited lower success rates [15,16,17,18]. However, advancements in surface modifications, the optimization of macro-geometry, and improvements in surgical techniques have significantly enhanced these success rates, resulting in their more frequent application by dental professionals. 

The nature of the implant–abutment connection also plays a critical role in determining success rates; implants that utilize internal Morse cone connections demonstrate a more uniform stress distribution in comparison to those featuring external hexagonal connections [19,20,21]. The Morse cone connection facilitates cold welding through friction between the abutment and implant surfaces, which minimizes micro gaps and consequently reduces bacterial infiltration [22,23]. The integration of the conical connection with short implants has resulted in a balanced distribution of loads across the implant, abutment, and surrounding alveolar bone [24,25]. 

To achieve a high success rate, it is crucial to establish a favorable biomechanical environment that enables the effective distribution of masticatory loads to the surrounding tissues. In this regard, finite element analysis (FEA) serves as a valuable tool for simulating the behavior of the implanted device within the bone, allowing researchers to model dental implants and bone interactions with increased accuracy and efficiency, thus mitigating the challenges associated with in vivo testing [16,26,27,28,29,30,31,32]. Specifically, the assessment of von Mises stress distribution serves as a crucial indicator of the stability of dental implants and the integrity of surrounding bone, both of which directly influence the longevity and long-term success of implant procedures. Indeed, the biomechanics of the chewing process generate axial and bending forces that produce a series of moments transmitted to the dental implant and subsequently to the surrounding bone. In this regard, several factors are instrumental in influencing the distribution of bone stress, which is pivotal to ensuring stability. For instance, excessive stress can lead to bone resorption and potential implant failure [33]. According to Frost’s theory [7], bone can initiate growth and remodeling processes in response to biomechanical demands based on the intensity and direction of applied stress. Moderate mechanical stress may stimulate bone remodeling, thereby enhancing bone density and strength, while also promoting Sema3A secretion and osteocyte proliferation. Conversely, excessive stress may induce pathological damage, as illustrated in Table 1 and discussed in a study by Uto et al. [34].

The stress exerted on bone tissue is also influenced by the geometric characteristics of dental implants [35,36,37]. A FEA study indicated that an increase in the implant diameter significantly reduces stress, in contrast to modifications in the length of the implant [37]. Furthermore, research conducted by Baggi et al. [35] demonstrated that the length of the implant predominantly affects the distribution of stress within the trabecular bone, thereby enhancing its overall distribution. For instance, a study by Zupancic Cepic et al. [38] specifically explored the biomechanical interactions of short implants (measuring 4, 6, and 8 mm) in supporting fixed partial dentures within the posterior mandible. The findings revealed that splinted configurations exhibited superior stress distribution compared to non-splinted configurations. Additionally, the results indicated that oblique loading at 30° resulted in greater stress distribution than axial loading. This observation suggests that oblique forces may generate higher and broader stress patterns around implants, potentially increasing the risk of damage to both the surrounding bone tissue and the implants themselves. 

It is crucial to acknowledge that the bone type significantly impacts implant stability and stress distribution. Azcarate-Velàzquez et al. [36] conducted an FEA to assess the effect of bone quality on two different types of dental implants. Their analysis demonstrated that a decline in bone quality led to poorer stress distribution and the overloading of the cortical bone at the implant collar. Another FEA study by Liu et al. [39] illustrated that the application of short implants in atrophied edentulous mandibles considerably affects the stress distribution within the surrounding bone tissue, where peak stress values tended to increase as the bone quality diminished. The classification system established by Lekholm and Zarb in 1985 [40] served as the foundational framework for examining the impact of bone quality and cortical thickness in this context. This classification categorizes bone into four primary types, ranging from Type I, characterized by dense cortical bone with a substantial amount of trabecular bone, to Type IV, which consists chiefly of trabecular bone with minimal cortical bone. While Lekholm and Zarb’s classification is centered on the four most clinically relevant bone categories, it may limit the study of more complex cases. To address this limitation, Misch [41] proposed an alternative classification in 1997, encompassing six categories that consider bone density and morphology, as well as the proportions of cortical and trabecular bone. This revised classification facilitates a more nuanced evaluation of bone quality, particularly in cases of diminished bone quality. Nevertheless, since short implants are primarily situated in medium- to high-quality bone, the classification established by Lekholm and Zarb remains applicable. It is essential to clarify that Lekholm and Zarb’s classification does not directly account for trabecular bone density, as the radiographic techniques employed for classification, such as panoramic and periapical radiographs, do not provide detailed information regarding trabecular bone density [40]. While these radiographs can reveal the amount of bone present, they do not yield precise assessments of bone density, which necessitates advanced imaging techniques, such as computed tomography (CT) or micro-CT. Furthermore, Lekholm and Zerb’s classification is more practical than that proposed by Misch, as it also incorporates the surgeon’s tactile perception during the site preparation for implantation. 

The implantation process itself is a significant factor influencing the distribution of stress within both the bone and the implants. Research conducted by Morita et al. [42] has demonstrated that the inclination of dental implants can lead to a reduction in stress levels, with decreases of 8.4% under a 90° load and 9.7% under a 60° load when compared to a vertical implant. On the other hand, the use of an inclined abutment resulted in a stress reduction of 15.7% under a 90° load and 30% under a 60° load relative to a vertical implant with a straight abutment. 

Notably, the factors discussed thus far, including the quality of the bone, do not currently provide a definitive indication regarding the most suitable positioning system: either inclined implants with straight abutments (II) or straight implants with inclined abutments (IA). Conducting an FEA study comparing II and IA configurations holds significant clinical relevance within the field of implant dentistry, as it offers insights into how variations in implant angulation and abutment alignment affect biomechanics, stability, and clinical outcomes. The application of II and IA is particularly relevant in clinical scenarios characterized by limited bone volume or unfavorable anatomical structures. Patients frequently encounter challenges such as inadequate posterior bone support due to resorption, which complicates the placement of straight implants without extensive bone grafting. The employment of II, IA, or short implants can enhance prosthetic support by leveraging existing bone while reducing the necessity for additional procedures that may elevate patient morbidity [43]. Additionally, tilted implants or abutments may enable longer prosthetic extensions without the corresponding cantilever risks associated with straight implants, thus improving both prosthetic support and functionality. This advantage is clinically significant, as it may lead to the more effective restoration of masticatory function for patients missing considerable posterior teeth. Clinical outcomes suggest that treatments utilizing angled implants, whether II or IA, contribute to enhanced patient satisfaction by mitigating perceived prosthetic instability—a prevalent concern among individuals relying on traditional denture solutions [44]. A systematic review revealed no significant difference in implant survival and success rates between strategies employing angled implants and conventional techniques over medium- to long-term follow-ups [44]. This finding implies that both strategies may yield clinically comparable results for patients facing anatomical challenges.

Therefore, the objective of the present finite element study was to investigate the bone stress distributions induced by two short implants, considering various cortical thicknesses (0.5 mm, 1.5 mm, and 3 mm) and utilizing the ANSYS Workbench 2023 FEA software (R23^®^, Canonsburg, PA, USA). This study examined two implant configurations: II positioned at a 30° angle and 30° IA, both of which featured Morse cone connections. These specific configurations were selected for investigation due to the scarcity of studies in the literature that have simultaneously assessed the effects of implant inclination or abutment in relation to bone type for short implants. A limited number of studies have evaluated the impact of the inclination of short implants corresponding to the bone class for different implant configurations (IA and II). For instance, a study conducted by Anitua et al. [45] indicated that the inclination of the implant is a more significant determinant of bone stress than the implant length itself. Specifically, a 17° inclination doubled the von Mises equivalent stress within the bone tissue, while a 45° inclination resulted in a 1300% increase in stress. Therefore, an inclination angle of 30° was chosen for this study, as it serves as an intermediate position that allows for the observation of inclination effects without reaching extreme angles such as 45°, which could induce excessive stress or fail to represent common clinical scenarios [45]. 

The null hypothesis proposed in this study posits that neither the configuration type (IA or II) nor the bone type influences the stress distribution.

## 2. Materials and Methods

### 2.1. Modeling

**Figure 1:**
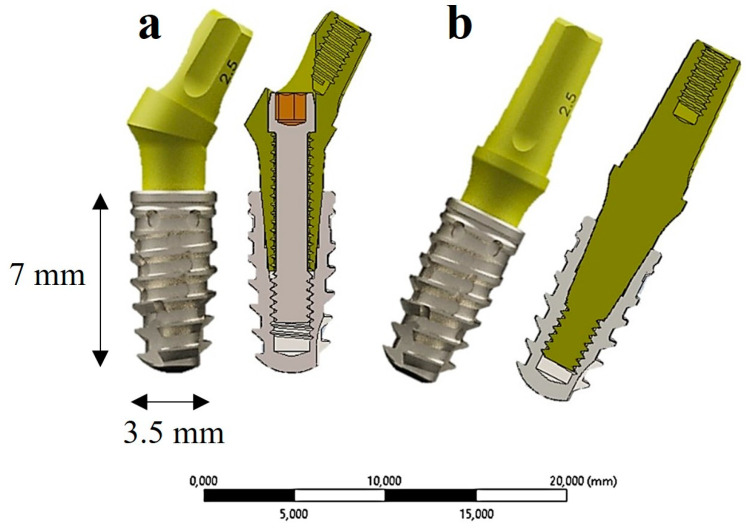
A three-dimensional (3D) model of the implants with abutments. (**a**) The implant with a 30° inclined abutment (IA); (**b**) the 30° inclined implant with a straight abutment (II).

The three-dimensional (3D) geometric models of the implants and associated components were procured from the implant company (Implacil De Bortoli, Sao Paulo, Brazil). These models were subsequently assembled utilizing computer-aided design (CAD) software (Autodesk Inventor 2023.1, San Francisco, CA, USA). Figure 1 represents an illustration detailing the short implants (Maestro) employed in this study. 

The implants in question possessed a diameter of 3.5 mm and a length of 7 mm. These dimensions were selected as they aligned with the designation of “short” implants as classified by the manufacturer (Implacil De Bortoli, Sao Paulo, Brazil). Additionally, the implants were engineered with healing chambers incorporated into their macro-geometric design to facilitate and expedite osseointegration, reduce bone compression during the insertion process, and improve the quality of the newly established bone tissue. Both implants are equipped with an internal Morse cone connection, which is intended to enhance implant stability and strength while mitigating the risk of micromovement and potential implant failure. Notably, the IA variant was designed with a Morse cone connection that includes a retention screw, whereas the II variant featured the standard implant–abutment Morse cone connection.

**Figure 2:**
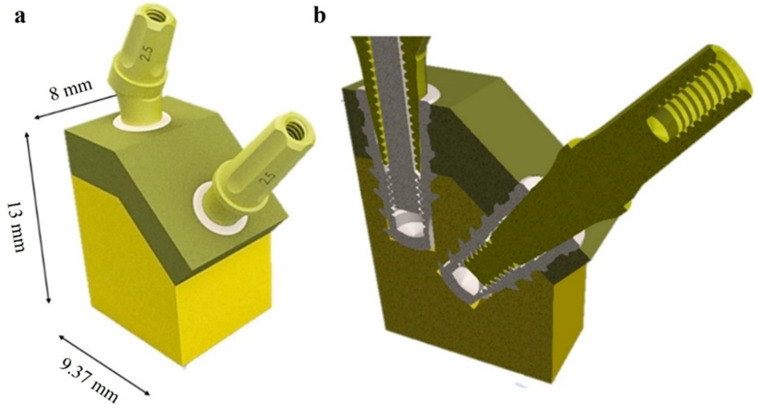
A 3D cortical and trabecular bone model. (**a**) The model with the inserted implants; (**b**) its sectional view.

Subsequently, the mandibular block model was constructed and designed according to the dimensions depicted in Figure 2. 

The 3D bone model was generated utilizing the same CAD software (Autodesk Inventor 2023.1, San Francisco, CA, USA) based on a CT scan [25]. Then, this model was employed to subtract the geometric thread profile following the insertion of the implant in a crestal position, ensuring a precise fit between the implant and the bone. It was assumed that, upon the insertion of the implant into the bone block, all surrounding bone surfaces are in contact with the outer surface of the implant. This assumption supports the concept of ideal osseointegration, thereby simplifying the modeling process. Full osseointegration indicates that the implant is stabilized through bony attachment, facilitating optimal load transfer during functional loading. In clinical practice, achieving successful osseointegration represents a fundamental objective in implant therapy, thereby proving a logical framework for FEA simulations that model the best-case scenario, wherein the implant functions as a unified entity with the bone. Utilizing simplified models is especially advantageous in exploratory studies, as it allows for an initial understanding of stress behavior without imposing excessive demands on computational resources.

### 2.2. Materials

A modification of the classification proposed by Lekholm and Zarb has been documented in previous research studies [46,47,48], confirming its validity through histomorphometric analysis, the measurement of bone mineral density (BMD), and micro-CT variables. For example, Pereira et al. [49] identified a correlation between the classification by Lekholm and Zarb and various histomorphometric parameters of bone.

**Figure 3:**
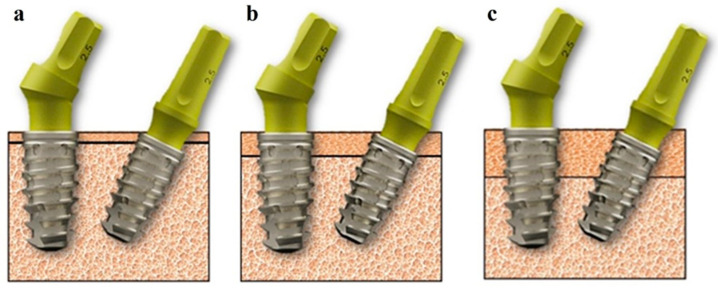
The cortical thickness for the three bone types studied with IA and II. (**a**) Type IV bone—cortical thickness: 0.5 mm; (**b**) Type III bone—cortical thickness: 1.5 mm; (**c**) Type II bone—cortical thickness: 3 mm.

Bergkvist et al. [50] calculated the BMD using Hounsfield Units (HU) obtained from micro-CT scans and observed a significant correlation between BMD and Lekholm and Zarb’s classification [48,50]. This classification assesses bone density and cortical bone thickness measured in HU, categorizing bone into three distinct types based on the density and thickness of the cortical bone:

- Case 1 corresponds to a Type IV bone, characterized by bone density ranging from 400 to less than 500 HU, with a cortical bone thickness of 0.5 mm encasing the spongy bone (Figure 3a);
- Case 2 represents a Type III bone, exhibiting a bone density ranging from 550 to less than 850 HU and a cortical bone thickness of 1.5 mm surrounding the spongy bone (Figure 3b);
- Case 3 pertains to a Type II bone with a bone density ranging from 850 to less than 1000 HU, featuring a cortical bone thickness of 3 mm encasing the spongy bone (Figure 3c) [48,50].

Bone serves as a prime example of an anisotropic material characterized by its heterogeneous composition and intricate microarchitecture. The mechanical properties of bone, including stiffness and strength, exhibit significant variability along different axes, attributable to its complex arrangement of cortical and trabecular structures. This inherent anisotropic nature results in distinct responses during compression and tension testing [51]. Furthermore, deformation and stress outcomes are influenced by the direction of loading. Non-invasive techniques, such as the integration of the vibrational response with CT or magnetic resonance imaging (MRI) data, hold promise for the development of more realistic bone models [52,53]. Bones are classified as cortical or trabecular, with the primary distinction resting in their mechanical characteristics; cortical bone is denser than trabecular bone. This difference in density is critical for assessing the bone’s capacity to support dental implants and to endure applied loads. Generally, denser bone correlates with greater stiffness and strength, which positively influences the stability of dental implants. Conversely, trabecular bone, characterized by a higher volume of internal trabecular structures, exhibits reduced density and, subsequently, lower mechanical properties [51,54].

To achieve accurate numerical results in FEA studies, it is imperative to model the properties of both biological and non-biological structures with precision. For instance, O’Mahony et al. [55] conducted a comparative assessment of the mandible utilizing fully isotropic and transversely isotropic (anisotropic) models. Their findings indicated that the anisotropic model yielded higher stress levels (up to 20%) compared to the isotropic model, particularly at the crestal region. However, there is a notable lack of studies in the literature that have thoroughly evaluated the anisotropic characteristics of mandibular and maxillary bone [56]. Consequently, in most studies present in the literature, the materials are represented with uniform, isotropic, and linear properties to simplify computational models [25,57,58,59]. 

The isotropic bone model utilized in this study serves as a foundational reference for comparisons with more complex models. Young’s modulus, as presented in Table 2, is a measure of the stiffness of a material and indicates the extent to which a material deforms under an applied load. A high value of Young’s modulus signifies that the material is stiff and experiences minimal deformation under stress [60]. The relationship between stress and Young’s modulus is articulated by Hooke’s law, which asserts that, within elastic limits, stress is directly proportional to deformation. Therefore, a bone with a higher Young’s modulus is capable of withstanding greater loads without significant deformation. According to Table 2, cortical bone exhibits a higher Young’s modulus (13.7 GPa) in comparison to trabecular bone (1.37 GPa), reflecting its greater density and stiffness. As a result, cortical bone is better equipped to support higher loads and withstand increased stresses compared to trabecular bone [25,57].

### 2.3. Finite Element Analysis (FEA) Simulation

**Figure 4:**
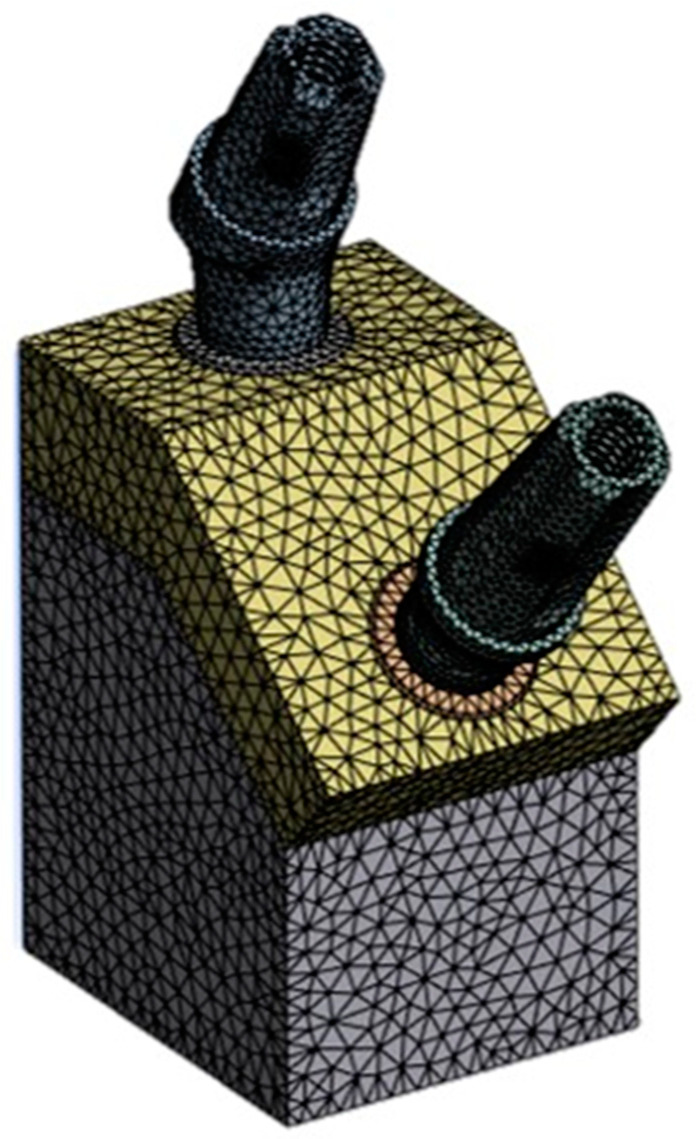
Schematic model of the mesh with IA and II configurations.

The 3D model CAD file, saved in .stp format, was imported into ANSYS Workbench 2023 FEA software (R23^®^, Canonsburg, PA, USA). The model was primarily discretized using 10-node tetrahedral elements (SOLID 187), with a maximum element size set at 0.5 mm (Figure 4) [25].

A sensitivity analysis was conducted to assess the influence of the mesh dimension on the results. Previous FEA studies have indicated that a mesh size of 0.5 mm yields reliable outcomes [25,57]. It has been determined that an element size of less than 0.3 mm is appropriate for accurately modeling the bone-implant interface [61]. Consequently, a mesh size of 0.3 mm was employed for the contact region between the implant and bone to enhance the estimation of the stress distribution. 

### 2.4. Loads and Constraints

**Figure 5:**
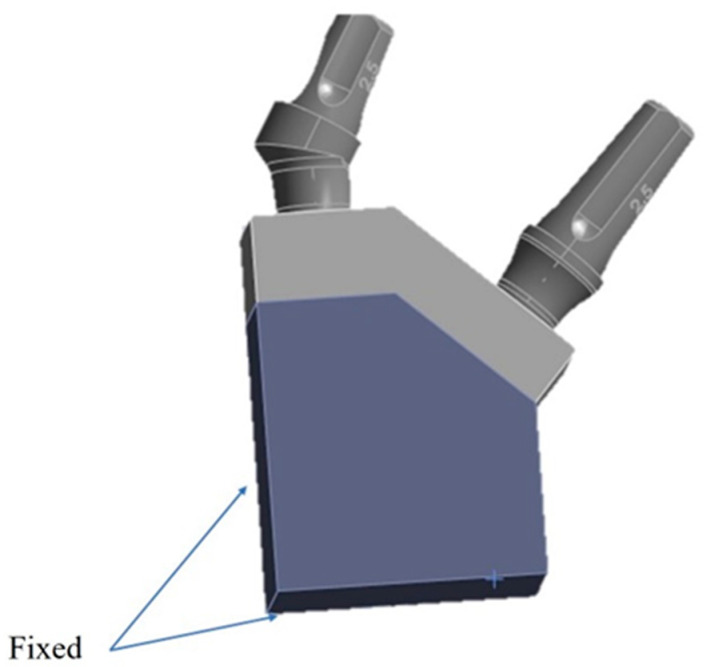
Constraint conditions.

The constraints established required the immobilization of the bone block in all spatial directions (Figure 5).

The implant was securely affixed to the bone, eliminating any potential for relative movement. Furthermore, a frictional contact was implemented on the outer surface of the abutment, utilizing a coefficient of friction of 0.3. However, it is important to note that the connection between the abutment and implant did not account for micro-movements or material fatigue at the interface.

**Figure 6:**
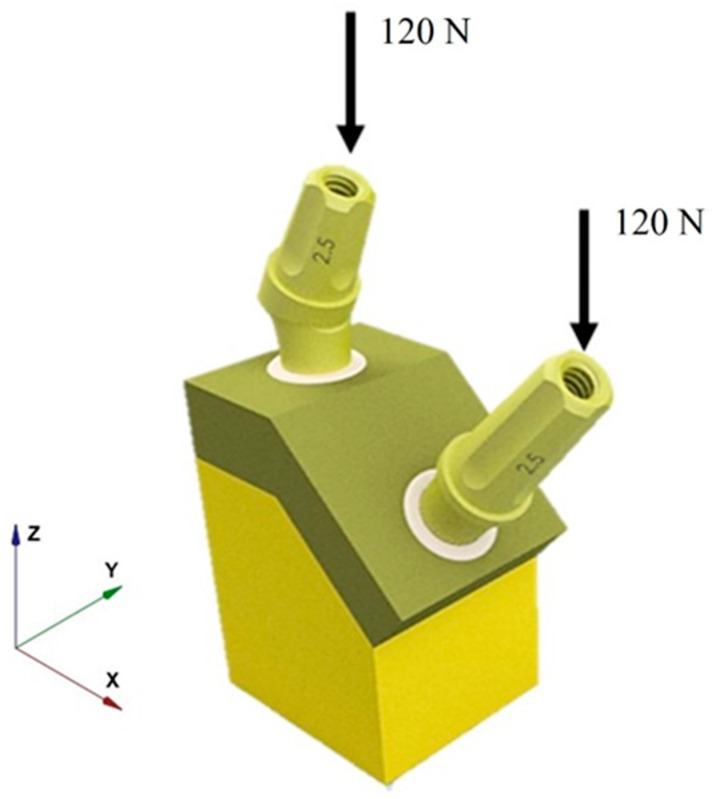
Application of a vertical load to the upper surface of IA and II configurations.

Molars are designed to withstand substantial vertical loads resulting from food compression and the activity of masticatory muscles during the process of chewing. Their morphology, characterized by prominent cusps and extensive masticatory surfaces, is optimized to accommodate these forces. Nonetheless, it is important to recognize that inclined forces, in addition to vertical ones, may also act on the molars, arising from lateral jaw movements or eccentric forces during the mastication of harder foods. Despite this, for the purposes of this study, the relatively low intensity of oblique loads, in comparison to vertical loads, was regarded as negligible [62]. A compressive force of 120 N was applied to the upper surface of the abutment along its long z-axis direction, simulating the forces generated during mastication on molars [42] (Figure 6).

The application of a vertical force induces a moment that generates mechanical stress in both cortical and cancellous bone, which can lead to deformations and an increased risk of fractures, particularly in regions with compromised bone quality. The primary distinctions between the IA and II configurations pertain to the fulcrum and the corresponding lever arm. In the case of the II, insertion is achieved by drilling a hole that is inclined at an angle of 30° relative to the surface of the alveolar ridge. The bending resulting from masticatory forces utilizes the apex of the implant as its fulcrum, thereby creating a lever arm that is greater than that of the IA, which operates with the fulcrum located at the Morse cone connection. This enhanced lever arm may contribute to elevated bending moments and, consequently, increased stress on the implant.

### 2.5. FEA Simulation

All models were analyzed on a Windows 10 64-bit system equipped with an Intel i7 processor and 16 GB of RAM. Following the analysis, the numerical results were converted into visual representations utilizing color maps. These color maps range from blue, indicating areas with the least stress, to red, which signifies areas of maximum stress. The assessment of stress levels was conducted using the von Mises criterion, which identifies the failure of a material when the equivalent stress exceeds its tensile strength. This criterion is instrumental in analyzing the transfer of loads from the implant to the surrounding bone. Previous FEA studies [63,64] have demonstrated that the stress concentration in particular regions can lead to bone resorption, thereby jeopardizing implant stability. Moreover, the distribution of von Mises stresses is influenced by factors such as the quality and thickness of the bone, whether cortical or trabecular [65]. Understanding these variations is essential for selecting the most suitable type of implant and surgical technique, which in turn promotes optimal bone integration and enhances overall health.

## 3. Results

### 3.1. Analysis of Stress on Implants

**Figure 7:**
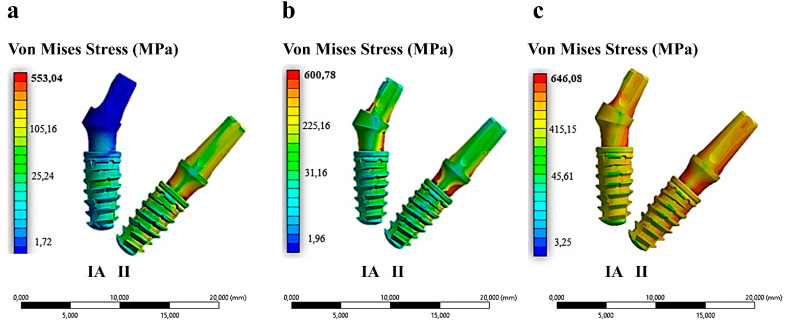
Von Mises stress distribution in implants placed in three different bone types and cortical bone thickness configurations. (**a**) Type II bone—3 mm; (**b**) Type III bone—1.5 mm; (**c**) Type IV bone—0.5 mm.

The research examined the mechanical performance of IA and II configurations in three distinct types of bone, each exhibiting varying cortical thicknesses, under a vertical load of 120 N (Figure 7).

As illustrated in Figure 7, the II configuration exhibited higher stress values compared to IA across all three bone types. Conversely, the IA configuration demonstrated elevated stress values solely at cortical thicknesses of 1.5 mm and 0.5 mm. For all implant types, the maximum stress occurred at the abutment neck, where the bending resulting from vertical chewing loads is more pronounced. Table 3 presents the numerical values of the stresses recorded at the neck of the implant for the three configurations of cortical bone thickness.

### 3.2. Analysis of Stress on Bone with the Implant Featuring an Inclined Abutment (IA)

**Figure 8:**
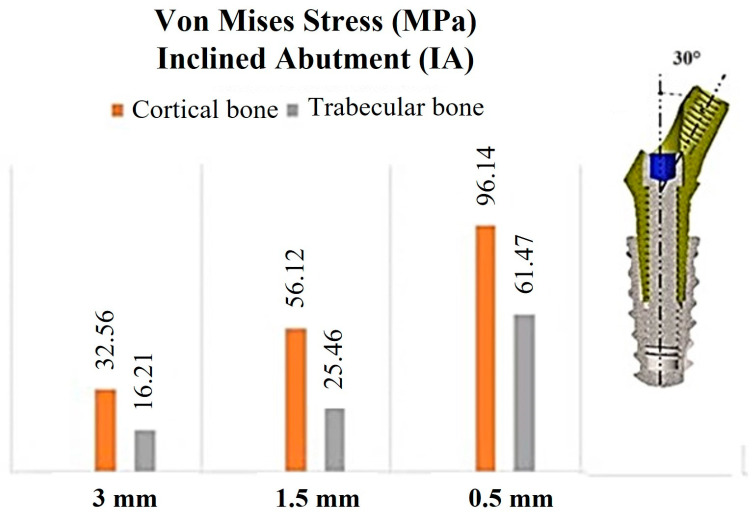
Von Mises stress values associated with the cortical and trabecular bones, in relation to cortical thicknesses of 3, 1.5, and 0.5 mm, with a 30° IA.

In Figure 8, the distribution of stress is presented for the implant exhibiting a 30° IA.

The decline in bone quality and the reduction in cortical thickness resulted in an increase in overall stress. It is evident that the stress on the implant is substantially higher than that on the surrounding cortical and trabecular bone, as illustrated in Figure 8. This phenomenon, referred to as “stress shielding”, is commonly observed in cases where a more rigid material is integrated into a less rigid substance, such as bone [66]. The rigidity of the implant, approximately 110 GPa, greatly exceeded that of cortical bone at 13.7 GPa and trabecular bone at 1.37 GPa. This disparity in rigidity results in the implant shielding the bone from stress distribution, thus creating a non-uniform force distribution between the implant and the bone. Furthermore, bone exhibits viscoelastic properties that enable it to absorb and dissipate certain stress levels. In contrast, the rigid implant tends to accumulate more stress, particularly in the neck region. While the higher stiffness of cortical bone (13.7 GPa versus 1.37 GPa for trabecular bone) protects the trabecular bone from excessive stresses, it leads to increased stress concentrations on the cortical bone. At a cortical thickness of 3 mm, the stress on the cortical bone surrounding the implant was measured at 32.56 MPa; at a 1.5 mm thickness, this stress increased to 56.12 MPa, and with a reduction to a 0.5 mm thickness, the stress tripled to approximately 96.14 MPa. This trend of heightened stress correlated with decreased cortical thickness was also prevalent in trabecular bone. For instance, at a thickness of 3 mm, the stress recorded was 16.21 MPa, which rose to 25.46 MPa at 1.5 mm, and at 0.5 mm, the maximum stress value reached 61.47 MPa.

### 3.3. Analysis of Stress on Bone with the Inclined Implant (II)

**Figure 9:**
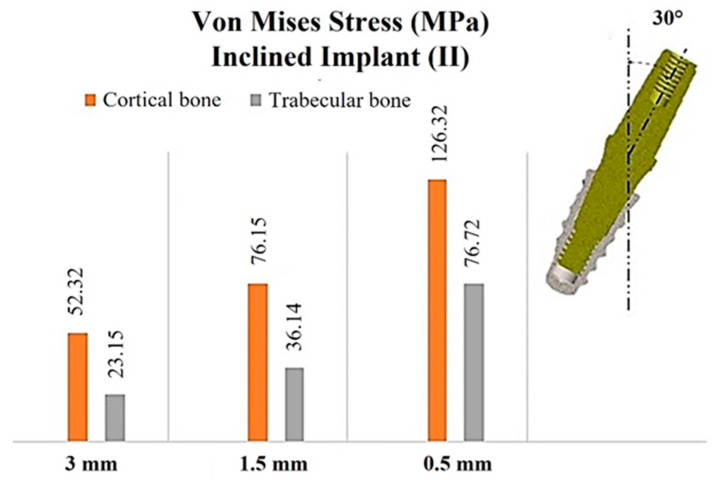
Von Mises stress values associated with the cortical and trabecular bones, in relation to cortical thicknesses of 3, 1.5, and 0.5 mm, with a 30° II.

The placement of the implant at an angle of 30° in the bone resulted in an increase in von Mises stress compared to the IA configuration (Figure 9).

For a cortical thickness of 3 mm, the stress exerted on the cortical bone was approximately 52.32 MPa. In contrast, with a thickness of 1.5 mm, the stress increased to 76.15 MPa, and at 0.5 mm of cortical bone, the stress reached critical levels of approximately 126.32 MPa. Given that the strength of cortical bone is estimated to be around 110 MPa [67], the latter scenario may precipitate significant concerns such as bone fracture. 

**Figure 10:**
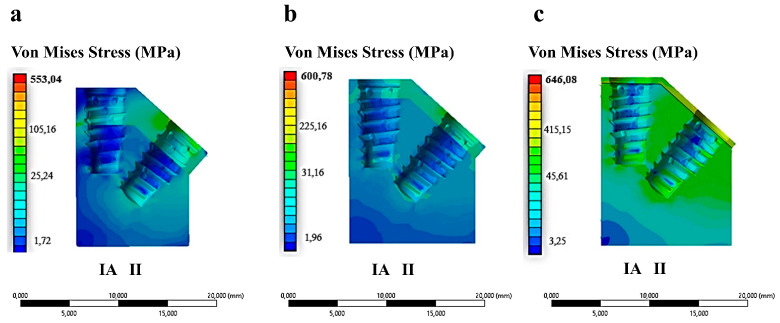
Von Mises stress distribution on IA and II as a function of cortical thickness: (**a**) 3 mm; (**b**) 1.5 mm; (**c**) 0.5 mm.

Furthermore, a substantial difference in the stress distribution on the trabecular bone was observed when comparing the positioning of an IA to that of an II. The positioning of the II resulted in a more uniform stress distribution across the trabecular bone. Additionally, the von Mises stress values recorded were 23.15 MPa within the trabecular bone for a cortical thickness of 3 mm, 36.14 MPa for a cortical thickness of 1.5 mm, and 76.72 MPa for a cortical of 0.5 mm. Figure 10 demonstrates that, with the II placement, a greater volume of trabecular bone was subjected to stress, with peak stress observed around the apex of the implant.

**Figure 11:**
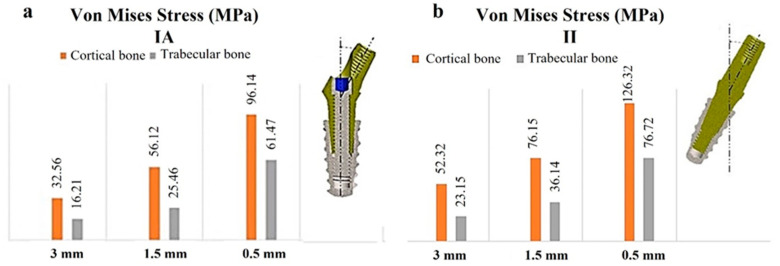
Von Mises stress values associated with cortical and trabecular bones in relation to cortical thickness. (**a**) IA; (**b**) II.

Figure 11 presents a comprehensive summary of the primary findings related to the distribution of stress in both cortical and trabecular bones. 

The left side of Figure 11 depicts an IA configuration, while the right side presents a configuration with an II.

The results derived from the FEA revealed several significant insights:

- A reduction in cortical thickness resulted in increased stress on both trabecular and cortical bone, particularly at the apex and neck regions, respectively;
- The II configuration exhibited higher stress levels compared to the IA; however, it also facilitated a more uniform stress distribution across the trabecular bone;
- The highest levels of stress were recorded within the cortical bone rather than the trabecular bone, especially for the II configuration at a cortical thickness of 0.5 mm, where stress reached 126.32 MPa. This value surpassed the maximum strength threshold of cortical bone (approximately 110 MPa), potentially leading to significant bone damage. In contrast, the stress for the IA configuration was measured at 96.14 MPa;
- Both implant insertion types did not present any complications for the implants, as the stresses encountered at the neck level remained below 900 MPa, which is acknowledged as the maximum strength capacity of titanium.

To mitigate the risk of bone resorption or fracture, the utilization of IA may be advisable, particularly in cases characterized by poor bone quality and thin cortical bone (approximately 0.5 mm). Conversely, when the quality of bone is deemed satisfactory and the cortical thickness exceeds 0.5 mm, an II can enhance the stress distribution across both cortical and trabecular bone, thereby decreasing the likelihood of bone resorption due to inappropriate bone stimulation. Additionally, the selection of the implant diameter is of paramount importance. An increase in the diameter by 0.5 mm corresponds to a 10–15% enhancement in the functional surface area. This factor is especially relevant, as the crestal region experienced the highest levels of stress, indicating that the implant diameter plays a more significant role than the length in mitigating these stresses. 

## 4. Discussion

The success of implant treatment is contingent upon the effective distribution of stresses to the supporting bone [68]. This distribution is influenced by several factors, including the implant geometry, bone type, type of loading, prosthetic design, as well as the healing and osseointegration processes [69]. Research has demonstrated that increasing the diameter of an implant diminishes the stress experienced by both the implant and the surrounding bone [24,35]. This reduction in stress is attributed to a more favorable distribution of forces across a larger functional area, which ultimately enhances stability [70,71,72]. Additionally, a larger implant diameter can mitigate bone loss by optimizing stress distribution at the implant collar [73]. Nonetheless, it is essential to acknowledge that excessively large diameters necessitate a substantial volume of bone, which may be in short supply due to bone atrophy, a prevalent condition resulting from tooth loss. The absence of a load on the teeth contributes to the resorption of bone in the mandible and maxilla [74]. This phenomenon was first described by Atwood in 1963 and was subsequently developed by Cawood and Howell in 1971, who categorized atrophy into six stages based on the quantity of residual bone available [75]. To address the challenges posed by bone atrophy and to facilitate the use of prostheses in affected patients, various techniques have been employed, including bone grafts, sinus lift, and guided bone regeneration, among others [76,77,78]. In cases of severe maxillary atrophy, short implants are frequently utilized when the quantity of available bone is limited. The application of short implants provides several advantages, such as the potential to circumvent more invasive and costly bone grafting procedures, a reduction in healing time, as well as a decrease in the risk of complications. Furthermore, these implants can be positioned in anatomically critical areas with constrained space, thereby stabilizing fixed or removable prostheses [18,79]. 

The type of bone surrounding dental implants is a crucial determinant of osseointegration, implant stability, and load resistance. Numerous studies have investigated the characteristics of bone and the various factors that may affect its quality and density [68,79]. For instance, Yang et al. [30] conducted a study that analyzed stress distribution on anatomically customized dental implants across different bone densities. The researchers modeled four types of maxillary bone utilizing Lekholm and Zarb’s bone classification system [40]. Their findings underscored the significant influence of bone density on stress distribution at the surface of dental implants, revealing diminished density corresponding with elevated bone stresses. 

Additionally, an FEA study by Shabanpour Kasari et al. [65] explored how varying levels of osseointegration influence the biomechanical response of the bone surrounding dental implants and stress distribution. This investigation highlighted the effects of osseointegration on stress values in both cortical and trabecular bone around dental implants. The results suggested that improved osseointegration enhances implant stability and diminishes bone deformation, thereby reducing the risk of implant failure due to excessive loading. Furthermore, the study emphasized the importance of bone quality and cortical thickness in the response of the short implant and the associated risk of bone resorption, particularly in low-quality bone regions, such as the posterior mandible.

Moreover, Liu et al. [39] examined the effects of the implant inclination on the stress distribution and support structures, highlighting the significance of proper implant and abutment configurations in minimizing stress concentration and ensuring uniform stress distribution. Indeed, the utilization of IA is recommended in scenarios involving poor bone quality and diminished cortical thickness to mitigate bone stress. Conversely, II and IA are advisable for facilitating better stress distribution in high-quality bone with a thick cortical layer. Notably, when subjected to oblique loads, II may lead to stress concentrations at critical locations, resulting in higher maximum stress values compared to straight implants [39]. This phenomenon may compromise the durability and strength of support structures and elevate the risk of long-term implant failure. Certain studies indicated that stress in the platform of a tilted implant may increase by as much as five times compared to that observed in a straight implant [65,79,80,81]. Additional investigations have also addressed the influence of implant inclination on cantilever use. Guven et al. [82] discovered that employing tilted implants in conjunction with short cantilevers can decrease stress on cortical bone by minimizing the moment of force generated, thereby relieving stress on adjacent bone tissues. 

However, in a numerical study [42], the application of IA was found to be more effective than II in reducing stress concentration. This increased efficacy arises because the head of the IA is situated closer to the occlusal surface, which serves as the primary loading point. This proximity facilitates a more equitable distribution of stress during loading and diminishes the concentration in specific areas.

Other variables may also influence the stress distribution within the implant and surrounding bone. For instance, a study conducted by Yao et al. [83] assessed the maximum values of the micro-gap between the implant and the abutment, which can serve as an indicator of the stability of the connection during loading. Specifically, a larger micro-gap may result in undesirable micromovements, negatively impacting the health of surrounding tissues and the longevity of the implant by creating localized stress concentration points where material failure is more likely to occur. Furthermore, the bending and torsional forces characteristic of mastication were shown to contribute to a deterioration in abutment stiffness, leading to progressive damage and an increased long-term risk of failure. In the present study, the application of the finite element method facilitated an examination of the mechanical response associated with two short implants positioned in two specific configurations (IA and II, both at an angle of 30°). This investigation encompassed varying cortical bone thicknesses and different bone types. 

It is essential to recognize that bone is a dynamic tissue, adapting its structure in response to external stresses. Numerous studies have established two critical stress thresholds pertinent to bone: one associated with resorption and another linked to fracture [84,85]. The findings from this study indicated that the II configuration with a cortical thickness of 0.5 mm exhibited significant stress levels on the cortical bone, measuring 126.32 MPa. Consequently, the risk of bone fracture under these conditions is considerably greater when compared to a cortical thickness of 1.5 mm (76.15 MPa) or to another thickness of 0.5 mm (52.32 MPa). Thus, the null hypothesis posited in the previous analysis may be appropriately rejected. 

Therefore, based on the outcomes of this research, the utilization of a 30° IA configuration is recommended in scenarios characterized by very thin cortical bone. Clinically, when bone quality is assessed to be high and the cortical thickness exceeds 0.5 mm, the placement of a 30° II configuration is advisable. Conversely, the IA configuration serves as a suitable solution across all categories of the bone class, as no stress values exceeding 120 MPa were recorded on the bone, while the stress on the implant itself measured 602.12 MPa, remaining below the strength threshold of the titanium alloy.

The results obtained, consistent with the existing literature, suggested that the implementation of straight implants and IA configurations is more advantageous in situations where bone quality is deficient. This is attributed to the reduced bone stress associated with this configuration compared to the II placement. This phenomenon can be elucidated by the hollow structure of the IA designed to accommodate the retention screw, which enhances stress dispersion. This characteristic effectively mitigates the stress concentration, promoting a more uniform distribution of stress throughout the connection and preventing areas of overload at the implant neck that could lead to bone resorption. 

Concerning the limitations of this study, it is important to acknowledge that finite element models serve as a simplification of the actual structures they represent. Consistent with previous literature, this study assumed that bone is homogeneous and isotropic to facilitate the modeling process. Such assumptions, however, may lead to considerable underestimations of stress distributions, thereby potentially compromising the accuracy and reliability of clinical outcomes [58,59]. Furthermore, a fixed contact between the bone and implant was assumed to simplify the analysis; yet, from a clinical perspective, achieving perfect osseointegration is implausible due to the inherent micromovements present at the implant interface [86]. Nonetheless, as delineated in a study by Kulkarni et al. [87], enhanced osseointegration is associated with increased implant stability and minimized bone deformation. The rationale for this assumption is derived from the clinical focus on attaining optimal mechanical stability and effective load transfer from the implant to the adjacent bone. Regarding load applications, it should be noted that these forces are time-varying and incorporate oblique components. For the purposes of this study, the oblique force components were neglected, as they are deemed to be less significant than the masticatory loads placed on molars. Additionally, the application of the load was simplified by directing force to the abutment, without accounting for the intricacies of the prosthetic crown. The failure to consider micro-movements and material fatigue at the abutment–implant interface may also represent a limitation, given that these factors can influence long-term stress distribution and overall implant success. A clinical investigation [83] has examined various phenomena pertinent to dental implant connections, including micro-movements and material fatigue. Such micro-movements at the implant interface could induce increased material wear and fatigue, thereby heightening the risk of mechanical failure and the subsequent loss of osseointegration, which could adversely affect the functionality of the implant. Nonetheless, tapered designs have demonstrated greater resistance to fatigue due to their capability to distribute forces evenly and mitigate micro-movements [88]. However, it is critical to note that should fatigue exceed a certain threshold, it may still lead to the fracture or failure of the connection, ultimately rendering the implant ineffective. 

Future research will aim to evaluate the impact of these limitations on numerical results to enhance the modeling accuracy, alongside the integration of necessary in vitro and clinical trials. Future investigations should specifically assess the effects of modeled bone anisotropy by incorporating mechanical properties derived from patient histomorphometric data. Additionally, both oblique and time-varying loads should be applied based on data collected directly from patients, thereby augmenting the proposed numerical analysis and facilitating validation through forthcoming in vitro and in vivo mechanical testing. By utilizing this information, clinicians may be better equipped to select optimal implant placement strategies based on anatomical variables and clinical considerations. 

## 5. Conclusions

In conclusion, the results of this study indicated that enhancing bone quality and cortical thickness is associated with a reduction in stress within the bone structure, provided that the system is not overloaded. Furthermore, the utilization of an IA, as opposed to employing an II, demonstrated a more significant reduction in stress, particularly in cortical bones with a thickness of 0.5 mm. As previously discussed, the fulcrum of bending generated by masticatory forces in the IA configuration was located at the Morse cone connection. This geometric arrangement facilitated an optimal load distribution between the implant and the surrounding bone. In contrast, with the II configuration, the fulcrum was positioned at the apex of the implant, which may lead to increased deflection in response to external forces. 

Therefore, in cases where bone quality is compromised and cortical thickness is diminished during implant placement, it is advisable to favor an IA configuration. This methodology alleviates stress on the cortical bone, thereby reducing the likelihood of bone resorption due to overload. A comprehensive understanding of stress distribution within bone structures can assist clinicians in selecting the most appropriate implant placements, taking into account anatomical variables and clinical factors. Further validation through in vitro and in vivo testing is essential to enhance the precision of the numerical analysis conducted in this study.

## Figures and Tables

**Table 1:** Bone behavior according to Frost’s theory [7].

Bone Stress	State of Bone
<1–2 MPa	Bone stops growing and resorbs
1–2 MPa	Bone remodeling may occur, but the bone mass remains unchanged
20–40 MPa	Bone remodeling occurs, and bone mass increases
40–60 MPa	Bone remodeling and bone mass significantly increase
>60 MPa	There is an overload with potential risks of resorption
120 MPa	The bone may break due to a fracture

**Table 2:** Mechanical properties of the materials used [25,57].

Component	Young’s Modulus (GPa)	Poisson’s Ratio
Abutment/implant (Ti6Al4V)	110	0.35
Cortical bone	13.7	0.3
Trabecular bone	1.37	0.3

**Table 3:** Von Mises stress values in implants placed in three cortical bone thickness configurations evaluated at the implant neck.

	Von Mises Stress (MPa)	
Cortical Thickness	Inclined Implant with a Straight Abutment (II)	Implant with an Inclined Abutment (IA)
3 mm	533.04	65.12
1.5 mm	600.78	576.48
0.5 mm	646.70	602.11

## Data Availability

The original contributions presented in the study are included in the article and further inquiries can be directed to the corresponding author.
